# Do topography and fruit presence influence occurrence and intensity of crop-raiding by forest elephants (*Loxodonta africana cyclotis*)?

**DOI:** 10.1371/journal.pone.0213971

**Published:** 2019-03-22

**Authors:** Steeve Ngama, Jerome Bindelle, John R. Poulsen, Jean-Luck Hornick, Annick Linden, Lisa Korte, Jean-Louis Doucet, Cédric Vermeulen

**Affiliations:** 1 TERRA Teaching and Research Centre, Forest Is Life, Gembloux Agro-Bio Tech, University of Liège, Gembloux, Belgium; 2 Laboratoire de santé et production animale, Département de Zootechnie, Institut de Recherches Agronomiques et Forestières, Centre National de la Recherche Scientifique et Technologique (IRAF-CENAREST), Libreville, Gabon; 3 AgroBioChem/TERRA, Precision livestock and nutrition unit/Agriculture Is Life, Gembloux Agro-Bio Tech, University of Liège, Gembloux, Belgium; 4 Nicholas School of the Environment, Duke University, Durham, North Carolina, United States of America; 5 Fundamental and Applied Research on Animal and Health, Animal Production Department, Faculty of Veterinary Medicine, University of Liège, Belgium; 6 Surveillance Network of Wildlife Diseases in Southern Belgium, Faculty of Veterinary Medicine, Liege, Belgium; 7 Independent Biodiversity and Natural Resources Specialist, Saint Cloud, MN, United States of America; University of Sydney, AUSTRALIA

## Abstract

Crop damage by forest elephants (*Loxodonta africana cyclotis)* and the resulting human-elephant conflict are issues of great concern for both the conservation of the species and the protection of rural livelihoods in Central Africa. Addressing these problems requires identifying the factors that facilitate or impede crop-raiding by forest elephants. Yet to date, the environmental or anthropogenic factors that influence the occurrence and intensity of crop-raiding by forest elephants are largely unknown. We used a multivariate approach to investigate conditions under which forest elephants raid some fields and not others in the buffer zone of Monts de Cristal National Park (MCNP), Gabon. We first interviewed 121 farmers from 11 villages situated within 10 km of MCNP regarding the occurrence of elephant crop-raiding of their fields. We then collected data on 39 explanatory variables to characterize the agricultural fields. Of these, the most important predictors of elephant raid occurrence of crop damage were presence of fruit trees, elephant deterrents (scarecrows, fire, wire string fences and empty barrels), and field topography. We secondly assessed the effect of stage of crop growth, presence of fruit trees, field topography and presence of elephant deterrents on crop-raiding occurrence and intensity by counting raids and measuring areas of crop damage every week in 17 plantations over 19 weeks in the most elephant-impacted zone of the study area. We found that fruit presence and stage of crop growth led to more intense damage to crops, whereas local deterrents did not inhibit raiding events and crop damage by elephants. We report a tradeoff between non-timber forest products (NTFP) services and crop-raiding by elephants. We show for the first time that steep topography impedes elephant damage to crops with no raids recorded in fields with surrounding slopes greater than 25%. We discuss whether farming on steep fields could be used as a strategy for mitigating crop-raiding to favor human-elephant coexistence and enhance elephant conservation.

## Introduction

Central Africa holds the second largest block of tropical moist forest in the world and most of the remaining habitat for forest elephants (*Loxodonta africana cyclotis)* [1,2]. Within Central Africa, the heavily forested country of Gabon harbors 22% of the range and more than half of the extant population of forest elephants [1–3]. Forest elephant numbers have declined dramatically in the past decade, and elephant crop-raiding amplifies this trend and turns rural people against their conservation because of heavy losses endured by farmers [4–8].

Elephant crop-raiding has harmful consequences for both rural farmers and elephants. Elephants can destroy an entire year’s crop in a single intrusion into a plantation, threatening the livelihoods of rural farmers and slowing the economic development of local communities [9]. In northern Cameroon, Tchamba [10] reported that elephant damage to crops led to losses of roughly $40,000 to $75,000 per year. In Democratic Republic of Congo, Inogwabini et al. [11] estimated an annual economic loss per farmer of about 77% of the mean GDP per capita. In Gabon, interviewed farmers reported losing 45% of their crops annually to elephant browsing [9]. During a government sponsored culling of elephants, two people were killed by elephants in Cameroun [10]. In Tanzania, Tingatinga villagers stated that elephants are responsible for about 75% of human mortality related to wildlife [8]. Loss of lives, crops, money, effort and time fuels resentment of elephants that often leads to retaliation killings conducted officially by law enforcement officers or unofficially by local people [8–11].

Resentment of elephants is widespread and expected to increase in rural Gabon, where elephants raid crops despite seemingly abundant forest habitat and plentiful natural food resources [9,12,13]. Gabon is 88% forest covered with 11% of its land mass protected in 13 national parks and has a relatively small human population (6.7 people km^-2^)–attributes that have made it a stronghold for the conservation of forest elephants [14–16]. With shrinking oil production, the Government of Gabon is working to diversify the economy through the promotion of both local and industrial agriculture [17–18]. With the expansion of agriculture and the excursion of human activities into wild spaces, incidents of crop-raiding will certainly increase as could resentment of elephant conservation [8,9]. The problem of crop-raiding needs to be urgently addressed.

To reduce elephant damage to fields, we need to understand the circumstances under which elephants raid crops. By raiding crops, elephants acquire high-energy food resources [19,20], but they may also obtain secondary health benefits such as lower parasites loads than non-raiding elephants [21]. Crop consumption, therefore, may serve additional purposes than fulfilling energy needs, such as the acquisition of rare nutrients or plant secondary metabolites that enhance immunity or combat parasites [21]. Elephants are large, mobile generalist herbivores [22]. They inhabit a wide array of environmental conditions and vary considerably in habitat use, social behavior, movement patterns and food selection [23–26]. Hence, the incentives for crop-raiding are likely multidimensional and not fully understood–perhaps one of the primary reasons for the lack of effective mitigation strategies to crop-raiding [6,25].

The aim of this study is to investigate the conditions under which forest elephants raid crops. Many anthropogenic and environmental factors likely drive crop-raiding, but little is known about environmental impediments to crop-raiding. Therefore, we address two main questions: (i) what are the main drivers and impediments of crop-raiding that could be integrated in strategies of forest elephant conservation? (ii) how do these factors influence the occurrence and intensity of crop damage? In Monts de Cristal National Park (MCNP) in Gabon the landscape comprises steep topography and abundant vegetation with multiple tree species. Consequently, we suspect that topography and fruit tree presence strongly influence crop-raiding. We hypothesize that: (1) field topography and presence of fruiting trees will be among the main factors determining whether a field is raided; and (2) presence of fruiting trees in a field will increase and steep topography will decrease the occurrence and intensity of damage.

## Methods

**Fig 1:**
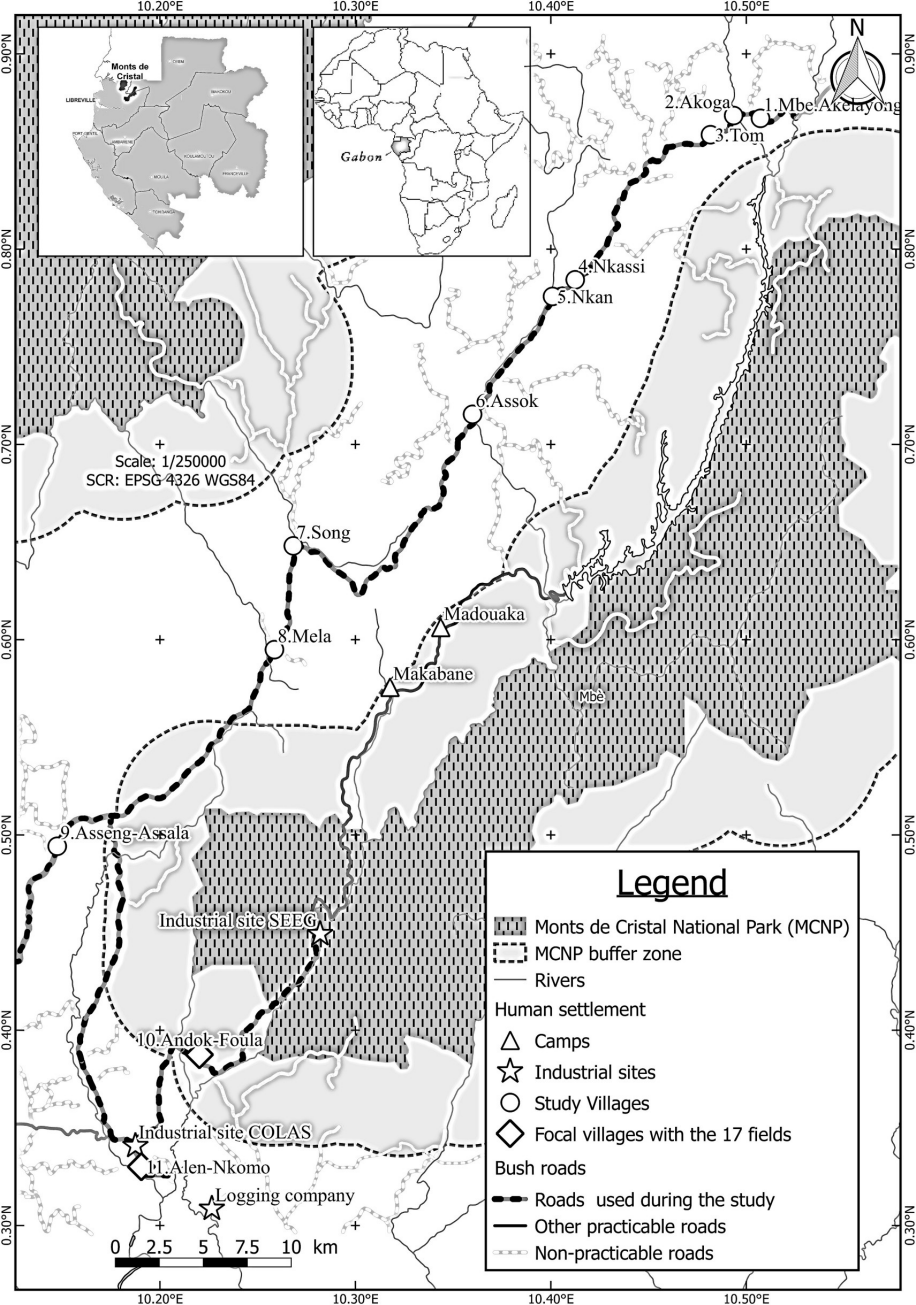
(Study site, TIF file): Mont de Cristal National Park (MCNP) and its buffer zones. With the exception of the southern part of the park, villages are located outside of buffer zones.

We conducted the study between June and November in 2014 and 2015 near human settlements in the southwest equatorial ecoregion of MCNP (1200 km^2^; Fig 1) [14,27]. MCNP is divided into two blocks by a 100 km road and 15 to 25 villages occur in the area between the blocks (Fig 1). Annual rainfall ranges from 2000 to 3500 mm, with two wet seasons: a short wet season occurring from September to December and a long wet season from February to May, interrupted by a long dry season from July to September that is moderated by cloud cover and a short dry season from December to January [14,27].

The park supports at least 3,000 plant species and is characterized by an abundance of *Aucoumea klaineana*, *Desbordesia glaucescens*, *Dacyrodes buettneri*, *Erismadelphus exul*, and plants from the genus *Bikinia* (syn. *Monopetalanthus*) [14,27]. Inselbergs, or rock outcroppings, support unique vegetation communities, including many endemic plants [27]. Wildlife in MCNP includes at least 35 species of mammals, 246 species of birds, 25 species of reptiles, 27 species of amphibians and numerous endemic butterflies [14]. Substantial populations of large mammals have been reported, including the western lowland gorilla (*Gorilla gorilla gorilla*), mandrill (*Mandrillus sphinx*), chimpanzee (*Pan troglodytes troglodytes*), buffalo (*Syncerus caffer nanus*), leopard (*Panthera pardus*) and forest elephant (*Loxodonta africana cyclotis)* [14]. We chose this study area because of the high incidences of crop-raiding from forest elephants, with reports of crop losses ranging between 6 and 8 on a scale of 0 to 9, with 0 signifying no elephant visits or crop damage and 9 indicating frequent crop-raiding events leading to abandoned fields [9].

The MCNP landscape is largely inaccessible to humans because of its dense forests and relatively steep topography (altitude ranges from 200 to 900 m) [14,27]. Consequently, human population density is low at 0.6 to 1.2 inhabitants per km^2^ [14,27]. Forests cover 97% of the landscape (65% of the area occurs in logging concessions, 18% in the park) and 3% is dedicated to traditional slash and burn agriculture (TSBA) [14,27]. In TSBA, trees and brush are cut to convert forest to agricultural land, and the resulting slash is burned to amend the soil [28]. Fruit-producing trees are left standing and serve as non-timber forest products (NTFP). In the study site, we recorded four NTFP tree species present in fields (S1 Table). The fruit of these species are known to be consumed by elephants [29,30]. Even though elephants consume fruit from a large number of tree species, we did not record other tree species commonly consumed by elephants nearby farms. Fruiting is seasonal, with the highest abundance of fruit at the transition between the wet season and the short dry season from December to February [29,31,32]. Some tree species produce fruit outside this period making fruit available year round [29,32]. In addition to fruiting trees, evergreen tropical rain forest is present in the study site and near the farms [14,27]. After 2 to 4 years of agriculture, farmers abandon old fields and restart the process [28]. In MCNP, crop species mainly consist of bananas (*Musa sp*.) and cassava (*Manihot esculenta*) followed by sweet potato (*Ipomoea batatas*), cocoyam (*Xanthosoma sagittifolium*), sugarcane (*Saccharum officinarum*), pineapple (*Ananas comosus*) and several types of vegetables (S1 Table) [14]. Our study sites consist of these traditional agricultural lands or plantations (i.e. crop fields), situated near villages along the dirt road and between the two blocks of MCNP buffer zones. The plantations were similar in crop composition as the region is inhabited by people sharing one traditional language (Fang), culture and agricultural methods.

Before data collection in the field, we received permission to conduct this research from: (i) the Centre National de la Recherche Scientifique et Technologique (CENAREST), permit number *AR0014/15/MESRS/CENAREST/CG/CST/CSAR*; (ii) the Agence Nationale des Parcs Nationaux (ANPN), permit number *4815010/PR/ANPN/SE/CS/AEPN*; and (iii) the Quality, Health Safety, and Environment (QHSE) Department of COLAS-Gabon. ANPN and CENAREST have the authority to grant permission to conduct research involving animals and humans in Gabon and within National Parks, while COLAS-Gabon is the main industrial company operating in the study site. The permission granting committees examined all methods and procedures. In addition, we used consent procedures for interviews that were reviewed and validated by an academic committee from the University of Liège. Fieldwork followed the relevant guidelines of these institutions.

We initially visited all villages situated within 10 km of the park borders and between the two park blocks to explain the purpose of the study to local authorities (traditional chiefs) and farmers and to obtain oral consent to work in their fields. We refer to these villages as “study villages” (Fig 1). During our initial visits, we recorded reports of elephant crop damage and people’s perception of the conservation of elephants and other animal species. We identified the period from June to November as the time period when (i) farmers record the most elephant crop damage, (ii) most crops are mature, and (iii) fruit tree species present near farms experience both fruiting and non-fruiting periods. We then conducted semi-structured interviews and surveys from June to November in 2014 and 2015. We combined semi-structured interviews and surveys to improve result accuracy.

Following established methods, we used semi-structured interviews along the 100 km road to assess the relative importance of explanatory factors of elephant crop-raiding in accordance with the first hypothesis [9,13]. Previous studies reported the highest incidences of crop-raiding in villages near MCNP [9]; therefore, we interviewed farmers from all study villages with 10 km of MCNP borders (Fig 1). Interviews involved 11 villages found in the study site, rather than the 15 listed on administrative maps (Fig 1). During the semi-structured interviews, we interviewed all farmers (N = 121) present in study villages. We gathered information on farmers, fields, farming practices and crops (S1 Table). We evaluated the effect of these 39 factors on three response variables: occurrence of elephant crop damage, occurrence rate of crop damage and farmer-estimated number of elephants that raided their plantations (number of elephants raiders) (S1 Table).

To assess elephant crop damage intensity and occurrence in relation to fruiting trees and topography, i.e., our second hypothesis, we surveyed all plantations (i.e. crop fields) in villages (‘focal villages’) that experienced the most crop-raiding events (Fig 1). We surveyed the 17 plantations once a week over 19 consecutive weeks from the end of June to the start of November. At each plantation, we measured the intensity of crop damage, the number of elephant raids (i.e. crop damage occurrence), and number of raiding elephants. We measured the intensity of crop damage with a meter tape after raids, determined the number of elephant raids from evidence of elephant intrusion (freshly damaged crops and elephants footprints) into fields, and estimated the number of raiding elephants by the number of differently sized footprints in fields. For explanatory variables, we recorded and identified all crop species in each plantation as well as their growth stages and the presence of elephant deterrents. We assessed topography of each plantation using a clinometer to measure the slopes of field borders, taking measurements at the four cardinal directions from the center of fields and classifying the topography based on the shallowest and steepest slope values. We defined three levels of field topography: flat fields (slope <10%), shallow fields (slopes from 10 to 25%) and steep fields (slopes >25%). Finally, we recorded and identified fruit-producing trees present in fields and estimated total plantation size with a handheld GPS (Garmin; Olathe, KS, USA).

Of the 17 plantations, eight were situated in flat fields (slopes <10%) and nine were located on hillsides (slopes >10%). Six plantations in flat fields were equipped with deterrents while none of the fields on hillsides were protected. We recorded fruit trees only in three plantations situated in flat fields and there were no obvious differences in crops planted in fields apart from their maturity (growth stage). Skid trails and bush roads passed within 10 m of the borders of ten plantations and the rest of the plantations were situated within 1 km of bush roads. Of the nine plantations situated on hillsides, two were on steep topography (slopes >25%) and seven on shallow topography (slopes of 10–25%). Yet, three of the seven shallow plantations had skid trails with walls more than two meters high on their shallowest sides. These skid trails created a steep topography adjacent to the three plantations. We thus included these three plantations in the steep topography group (N = 5). Of the flat fields, three contained fruit trees and six were equipped with traditional deterrents against elephants, such as scarecrows and wire fences. One farmer lived in his plantation to scare away elephants by banging on an empty barrel and building fires.

We were limited in the amount of information that could be gathered because the sample size was limited by the agriculture activities of the area. The 17 surveyed fields represented 100% of farms present during the study in the focal villages in the south MCNP, with many people claiming to have abandoned plantations because of elephant crop-raiding. We did not include farms from the other villages, which were subject to fewer elephant intrusions. We thus conducted investigations over five months rather than our originally scheduled two weeks. This allowed us (i) to perform a longitudinal investigation covering both fruiting and non-fruiting periods, and (ii) to increase the number of observations of crop-raiding to overcome the limited number of villages. Plantations contained the same crop species apart from a few leafy vegetables. We could not estimate crop density (crops ha^-1^) and/or the proportion of planted area (% of occupied area/crop) because multiple crop species were interspersed in plantations making it difficult to accurately measure them independently. Many elephant raids destroyed nearly all crop plants in fields, making it impossible to count the number of plants eaten or damaged or to identify those plants to species.

With the farmer interview data, we determined the relative importance of the 39 explanatory variables on the occurrence of elephant crop damage. We determined the occurrence rate of crop damage and number of elephant raiders and used them in the Classification and Regression Trees (CART) to discriminate among factors. CART presents data in a way that is easily interpreted and determines the most important variables based on their explanatory power [33]. The CART method enabled us to compete and discriminate factors with the most to least influence on elephant crop-raiding. For all CART models, we used backwards stepwise model selection to reduce the full model to the minimum adequate model [33–35].

With the field survey data, we assessed the effects of stage of crop growth, presence of fruit trees, field topography and presence of elephant deterrents on crop-raiding intensity (area of damaged fields), number of elephant raids and number of raiding elephants. Our dataset comprises both discrete and continuous data. We analyzed discrete data with non-parametric statistics and continuous data with parametric statistical tests (ANOVA and Tukey post hoc tests). We modeled the area of damaged fields using linear mixed models (LMM) and modeled the number of elephant raids and number of raiding elephants using generalized linear models (GLM). We chose those models because they allow different types of responses (continuous, discrete) to be modeled by a linear function of explanatory variables while appropriately describing how the variance depends on the mean [34,35]. When modeling the area of damaged fields, we treated weeks as a random effect in LMMs to account for temporal autocorrelation that could be associated with the fruiting state of trees and repeated visits by elephants. We also included plantation identity as a random effect to account for specific differences in sites related to topography, crop growth stages, deterrent presence, and presence of fruit. Because fruit trees only occurred in the three plantations equipped with elephant deterrents, we did not include presence of fruit trees as an explanatory variable to avoid multicollinearity. For the number of elephant raids and number of raiding elephants, we modeled the response variables using a Poisson or negative binomial distribution to account for over-dispersion [34,35]. For both LMMs and GLMs, the Akaike Information Criterion (AIC) was used to compete models and to determine the most parsimonious model. We additionally conducted ANOVA and Tukey post-hoc tests to assess differences in areas of damaged fields between fruiting and non-fruiting periods, plantations with and without fruit trees, plantations in flat, shallow and steep topography, and stages of crop growth. Finally, we performed Wilcoxon and Kruskal-Wallis rank sum tests to evaluate differences in numbers of elephant raids and numbers of raiding elephants between fruiting and non-fruiting periods, plantations with and without fruit trees, plantations in flat, shallow and steep topography, and between stages of crop growth.

We used R version 3.0.3 (R Development Core Team 2014) for all statistical analyses.

## Results

### Interview results: The main predictors of crop-raiding

In our study villages, we interviewed 121 farmers with 241 plantations (2±1 plantations per farmer). Of the 121 farmers, 53.7% were female, 58.7% of farmers were > 60 years old, average age was 50 years old and none were younger than 18 years old.

The three response variables were differentially affected by independent variables. According to farmers, the most important response variable is occurrence of elephant crop damage, followed by number of elephant raiders. Occurrence of crop damage per year was most important because a single visit can destroy an entire field. For occurrence of elephant crop damage, fruit tree presence had the strongest effect followed by field topography, type of deterrent, season and type of cropping activity (Table 1). For number of elephant raiders, fruit tree presence again had the strongest effect, followed by extent of damage on the main crop field, farmer revenue and deterrents. Occurrence rate of crop damage per year was predicted by season, distance of fields from park borders, and presence of the most preferred crop and deterrents (Table 1). Hence, presence of fruit trees, field topography and presence of elephant deterrents were among the main factors predicting whether a field would be raided or not in MCNP.

**Table 1:** CART results from farmer interviews.

Variable Ranks	Occurrence of elephant crop damage	Rate of crop damage per year	Number of raiding elephants
**1**	Fruit tree presence (121)	Seasons (56)	Fruit tree presence (121)
MIV	Yes = 1.96 (57)	MWS&MDS&SWS/ = 1.95(21)	No = 1.02 (64)
LIV	No = 1.01 (64)	All seasons = 3 (35)	Yes = 1.96 (57)
**2**	Field topography (121)	Distance of farms from MCNP border (56)	Extent of damage on the 1^st^ plantation (68)
MIV	Shallow & Steep = 1.1 (70)	>10km = 2 (22)	Total or major = 1.6 (33)
LIV	Flat = 1.96 (51)	<10km = 3 (34)	minor = 2.29 (35)
**3**	Type of main deterrents (121)	Distance of farms to MCNP buffer zone (56)	3^rd^ revenue source (68)
MIV	Type 1 = 1.13 (74)	>5km = 2 (22)	Agriculture = 1.13 (8)
LIV	Type 3 = 1.97 (47)	<5km = 3 (34)	Others = 2.03 (60)
**4**	Season 6^th^ task is implemented (121)	3rd most damaged crop (56)	Type of deterrents (68)
MIV	SDS = 1.14 (76)	Others = 2.14 (22)	Type 2 = 2.23 (30)
LIV	MWS&MDS = 2 (45)	Cassava and cocoyam = 2.91 (34)	Type 5 = 1.68 (38)
**5**	Type of 5^th^ implemented task (121)	Type of deterrents (56)	Implementation season of the 6^th^ task (68)
MIV	Others = 1.17 (79)	Type3 = 2.05 (26)	SDS = 1.57 (23)
LIV	Deterrent setting = 2 (42)	Type 4 = 2.97 (30)	MWS, MDS = 2.11 (45)

MIV = most influent sub level variable. LIV = Less influent sub level variable. Response variables are bolded in the first row, and explanatory variables are presented beneath them according to their relative importance (highest to lowest importance). The underlined terms are factors, and levels of each factor are listed below them with their regression coefficients and number of observations in parentheses. For example, ‘fruit tree presence’ is the most influential variable on occurrence of elephant crop damage (mean Yes = 1.96, n = 57; mean No = 1.01, n = 64). Types of deterrents are combinations of methods that change according to villages and farmers.

### Crop damage assessment results: Impact of fruiting tree presence and topography on elephant crop-raiding occurrence and intensity

The 17 plantations assessed for crop damage represented 38,975 m^2^ of crop area, with a mean area of 2,293 ± 1,938 m^2^ per plantation (Table 2). We recorded 180 elephant intrusions, approximately 11 ± 10 intrusions per field in 19 weeks (Table 2). About 75% of field area was damaged with a mean of 90.7 ± 53 m^2^ damaged per plantation per week (Table 2). Six plantations situated in flat topography and two in shallow topography were completely destroyed with 100% crop loss at the end of the study, whereas none of the five farms with steep topography were damaged or raided (Table 2). One plantation in flat topography and one in shallow topography were also never raided during the survey; however, the final two plantations were partially damaged. Two fruit trees species (*Pseudospondias microcarpa* and *Irvingia gabonensis*) were present in and around three plantations (with one or two trees present per plantation) and we recorded evidence of elephants eating the fruit of both species. Plantations were comprised of 50–60% manioc, 40–50% banana and less than 10% of other cultivated plants (S1 Table).

**Table 2:** Results from the survey of 17 plantations in two focal villages.

					Kruskal-Wallis and Wilcoxon tests				ANOVA			
Explanatory variables and their sub-levels	No. of fields	Field area (m^2^)			Number of elephant raids		Number of raiding elephants		Area of damaged crop fields (m^2^)			
		Total area	Mean/field		Mean number of raids per month	Test results	Mean number of raiding elephants per month	Test results	Total of damaged area	% of losses	Mean/field /week	Results
All fields	17	38,975	2,293±1,938		11±10	-	29±26	-	29,300	75	90.7 ± 53	-
Plantations with fruiting trees	3	8,300	2,767±1,567		1±0.8 ^a^	*W = 616*.*5*	5±4 ^a^	*W = 249*.*5*	5,780	70	193±183^a^	*F = 3*.*2*, *df = 1*
Plantations without fruiting trees	3	8,300	2,767±1,567		2±1 ^b^	***p<0*.*05***	6±5 ^b^	***p = 0*.*08***	1,020	12	37.8±32.8^b^	***p = 0*.*07***
Plantations with fruit trees	3	8,300	2,767±1,567		5±3 ^a^	*W = 4061*.*5*	6±5 ^a^	*W = 748*	6,800	82	119.3±109.3^a^	*F = 1*.*5*, *df = 1*
Plantations without fruit trees	14	30,675	2,191±1,891		1.3±0.9^b^	***p<0*.*05***	3±2 ^b^	***p<0*.*05***	22,500	73	85±47^a^	*p = 0*.*22*
Flat fields (<10%)	8	18,600	2,325		4±0.5 ^a^	*X^2^ = 111*.*41*	4±3 ^a^	*X^2^ = 0*.*005*	16,800	90	111±101^a^	*F = 6*.*7*,
Shallow fields (10 to 25%)	4	13,025	3,256		1±0.5 ^b^	*df = 1*,	4±3 ^a^	*df = 1*,	12,500	96	164±127^b^	*df = 2*
Steep fields (>25%)	5	7,350	1,470		0	***p<0*.*05***	0	*P = 0*.*9*	0	0	0	***p<0*.*05***
Deterrent present	6	11,800	1,967		4±3 ^a^	*W = 7590*.*5*	3±2 ^a^	*W = 2483*.*5*	10,000	85	88±50^a^	*F = 0*.*3*, *df = 1*
Deterrent absent	11	27,175	2,470		2±1 ^b^	***p<0*.*05***	5±4 ^b^	***p<0*.*05***	19,300	71	92±55^a^	*p = 0*.*6*
Vegetative & flowering crops	-	-	-		2±1 ^a^		5±4 ^a^		7,050	-	88.1 ± 38.1 ^a^	
Vegetative, flowering & fruiting crops	-	-	-		3±2 ^b^	*X^2^ = 17*.*2*,	7±5 ^b^	*X^2^ = 15*.*1*,	5,600	-	147.4 ± 97.4 ^a^	*F = 0*.*4*,
Vegetative, flowering, fruiting & senescent crops	-	-	-		2±1 ^a^	*df = 3*,	4±2 ^a^	*df = 3*,	9,225	-	80.9 ± 30.9 ^a^	*df = 3*,
Flowering, fruiting & senescent crops	-	-	-		2±1 ^a^	***p<0*.*05***	3±2 ^a^	***p<0*.*05***	7,425	-	81.6 ±30.6 ^a^	*p = 0*.*7*

ANOVA, Kruskal-Wallis and Wilcoxon tests demonstrate the effects of field topography, presence of fruit trees, elephant deterrents and crop growth stage on elephant crop-raiding behavior. Consecutive figures with different letters (a, b) are significantly different at 95% confidence level. All means are expressed per field (or plantation unit). Means were not used for Kruskal-Wallis and Wilcoxon tests calculations as they compare ranks, but means are presented for comparison.

In our LMMs, only growth stage of crops had marginally significant effects on area of damaged crops, with vegetative, flowering and fruiting crops tending to increase the area damaged compared to the senescent stage (Table 3). In our GLMs, the number of elephant raids was predicted only by field topography, with shallow topography having significantly lower numbers of elephant raids than flat topography (Table 3). The number of raiding elephants was predicted by field topography, presence of deterrents, and growth stage of crops. Steep topography and presence of deterrents significantly decreased numbers of raiding elephants, whereas mature crops (vegetative, flowering and fruiting crops) increased the number of raiding elephants (Table 3).

**Table 3:** Results of generalized linear (GLM) and linear mixed (LMM) models examining factors that best explain the elephant crop-raiding behavior.

		LMM					GLM							
		Crop field area damaged					Number of elephant raids				Number of elephant raiding			
		Est.	SE	df	*t*	*p*	Est.	SE	*z*	*p*	Est.	SE	*z*	*p*
	Intercept	70.3	120.5	12.6	0.6	0.57	0.05	0.29	0.17	0.86	1.89	0.18	10.4	**<0.05**
Field topography	Steep topography (>25%)	-149.5	105.7	12.6	0.6	0.6	-19.6	96.3	-0.02	0.9	-	-	-	**-**
	Shallow topography (10 to 25%)	35.3	121.4	10	0.3	0.78	-1.5	0.32	-4.6	**<0.05**	-0.54	0.17	-3.23	**<0.05**
Deterrent	Deterrent present	-32.7	114.8	10	-0.3	0.78	-0.33	0.25	-1.3	0.18	-0.85	0.15	-5.42	**<0.05**
	Vegetative and flowering crops	65.5	72.5	13.8	0.9	0.38	0.26	0.22	1.2	0.23	-0.06	0.14	-0.4	0.7
Growth stages of crops	Vegetative, flowering and fruiting crops	107.6	61.6	232.9	1.7	**0.08**	0.22	0.23	0.97	0.33	0.46	0.14	3.25	**<0.05**
	Vegetative, flowering, fruiting and senescent crops	151.8	117.3	11.4	1.3	0.2	0.5	0.34	0.15	0.14	0.002	0.21	0.01	0.99

Results are from 17 plantations in two focal villages. Est = estimate, SE = standard error; df = degrees of freedom, *t* and *z* are statistics, and *p* is the p-value. Fruit presence was not included because of the low number of observations. “Fruit tree presence” and “fruiting tree” variables were not included when fitting models to avoid multicollinearity with “deterrent presence” because fruit trees were present only in three plantations that were all equipped with deterrents.

When trees fruited in plantations, a significantly greater area was damaged than in plantations without fruiting trees (Table 2). Presence of fruit trees did not significantly affect the area of fields damaged, but the number of raiding elephants and elephant raids were higher in plantations with fruit trees (Table 2). Field topography significantly predicted the number of elephant raids and number of raiding elephants, with no crop raids taking place in plantations with steep slopes (Table 2). There were significantly more elephant raids in flat fields than shallow and steep fields, and higher crop area damage in plantations with shallow slopes than steep slopes (Table 2). The number of raiding elephants was not significantly different in plantations with shallow topography and flat fields (Table 2). Plantations with traditional elephant deterrents tended to have lower areas of damaged crops, but this difference was not statistically significant (Table 2). The number of elephant raids was higher in plantations with deterrents, while the number of raiding elephants was higher in plantations without deterrents (Table 2). The area of crop damaged in mature crops (vegetative, flowering and fruiting crops) was not significantly different than area damaged in other crop growth stages (Table 2). There were significantly higher numbers of elephant raids and numbers of raiding elephants in plantations with mature crops (vegetative, flowering and fruiting crops) than immature crops (Table 2).

In summary, steep topography decreased the intensity and occurrence of crop-raiding events. The effect of fruit trees was confounded by the presence of elephant deterrents, but the intensity and occurrence of crop-raiding events tended to increase during the fruiting period and when crops were mature.

## Discussion

With growing economic development planned in Central Africa and Gabon, rates of human-elephant conflict are expected to increase. For the moment, MCNP remains a wilderness with very low human population and few farms [27]. Recent data suggest that the rural population is declining [14] in accordance with our observations of abandoned villages. However, the rate of crop damage by elephants recorded during the survey was very high at 75% of total surveyed field areas (Table 2). Similarly high levels of crop damage were recorded in other regions of Gabon [9]. This is surprising given that in MCNP forests represent 97% of the landscape around the villages (Fig 1) [14,27]. With the livelihoods of rural people and conservation of elephants threatened by crop-raiding, the ability to protect fields from elephants emerges as a significant challenge [7,8]. In this study, we determined the drivers of elephant crop raid occurrence, report a tradeoff between non-timber forest products (NTFP) and crop-raiding by elephants and demonstrate, for the first time, that elephants do not raid fields with steep topography (slopes > 25%).

Of 39 environmental and anthropogenic variables recorded in interviews with rural farmers, topography, presence of deterrents, crop maturity and presence of wild fruit were most important for explaining crop-raiding by forest elephants. The presence of wild fruiting trees increased the occurrence of elephant crop damage, suggesting that elephants are attracted to farms for wild fruit and not just crops. Rural people in MCNP practice slash-and-burn farming, cutting down trees and standing vegetation, but they retain fruit-producing trees as NTFPs [30], specifically four tree species: *Pseudospondias microcarpa*, *Chrysophyllum africanum*, *Irvingia gabonensis* and *Tetrapleura tetraptera* (S1 Table).

Plantations in MCNP buffer zones attracted elephants when fruiting trees were present in the fields and when crops were mature. The presence of fruiting trees was the most important factor drawing elephants into fields (Table 2), even when farmers employed deterrent strategies. Presence of fruiting trees strongly influenced both the occurrence of elephant crop damage and the number of raiding elephants. Elephants also damaged more crop area during fruiting periods compared to non-fruiting periods (Table 2). Therefore, elephants distinguish between trees with and without fruit and are attracted by fruit [26,29,31]. Mature crops also attracted elephants into plantations, increasing both the area of crop damage and the number of raiding elephants (Table 3). These results suggest that elephants raid plantations for both wild fruit and crop foods in accordance with previous results [19,20,26,31].

With recent evidence of dramatic declines in forest elephant populations [4,5], new management strategies are needed to conserve the species, which is listed as vulnerable on the IUCN Red List [36]. The importance of productive agriculture for combating poverty and contributing to wildlife preservation is well established [37–39]. Yet, crop-raiding impedes farming productivity and inspires resentment against elephants [8]. One important step towards elephant conservation, therefore, would be to reduce crop-raiding [7,40]. Crop-raiding might be reduced by retaining fruit trees outside of fields, rather than in the interior.

Elephants create trails to fruit trees [26,41]. Local farmers should be informed that fruiting trees attract elephants and encouraged to avoid farming near them. However, fruiting trees also attract seed dispersing animal species and can accelerate forest regeneration after field abandonment [23,42]. More detailed research is thus necessary to better understand movement patterns of elephants in relation to fruiting trees in farm lands and the tradeoff between NTFP services and crop-raiding.

Farming on slopes might also reduce crop-raiding by elephants. In MCNP, plantations on hillsides with shallow and steep fields had fewer raids than those on flat fields. MCNP has a highly variable topography that includes steep fields with slopes greater than 25% [27]. Our study appears to be the first assessment of elephant crop-raiding in steep topography. There were no crop-raiding events in plantations with slopes greater than 25% during our survey (Table 3). Elephants might not walk up steep slopes because of the energy costs, or they may just select flatter terrain when they have the choice [24,43]. In Kenya, Wall et *al*. [24] reported the importance of steep terrain in guiding the movements of savannah elephants. In Gabon, Terborgh et *al* [44] recently reported that elephants use vegetation differently on level ground compared to slopes: elephants did not forage on the steepest slope they investigated. This is in accordance with our findings that no crop raid events occurred on the steepest slopes. In their study, Terborgh et *al* [44] found a significant effect of slope on elephant plant selection with incidences of break scars on saplings being greater on level ground than on slopes [44]. Elephants are generalist herbivores that are first attracted by the quantity of forage rather than the quality [20,21]. In addition, animals climbing slopes must lift their body mass increasing the metabolic requirements for large animals [43,45]. Even small inclines represent barriers of movements for many large organisms [24,43,44,45]. During the survey, elephants successfully entered two plantations with shallow slopes (10 to 25%) and destroyed them, indicating that they can cope with slopes up to 25%, particularly when food is available. But elephants never entered plantations with steep slopes (>25%).

We identified at least two factors that influence crop-raiding by forest elephants, but crop type might also affect crop-raiding and damage. With traditional slash-and-burn agriculture, crops are mixed according to farmers’ personal perceptions of soil suitability making it difficult to accurately measure the composition of crop types. Even though our statistical results (CART) and personal field observations did not find evidence that crop type affects elephant crop-raiding, future investigations should address this issue. One possible solution would be to identify and count every individual plant in the fields being studied.

Testing the effectiveness of farming on steep slopes as a mitigation strategy for crop-raiding will require additional research. Our study was conducted within a limited time period of five months, and elephants might try to enter fields on hillsides after raiding those on flat and shallow topography. Future investigations should cover at least one crop production cycle of twelve months. Crop-raiding mitigation strategies could use steep slopes as an elephant deterrent, but implementation of the strategy would differ at small and large scales. At the small scale, the most effective strategy might be to incorporate topography with other deterrents, such as encircling fields on steep slopes with wire fences. Such methods could be practically implemented by rural people. On flat terrain where hillsides do not exist, dirt walls could be built around fields, mimicking the skid trail walls that were effective in deterring elephants in our study. This strategy likely requires support from industry or government because of the construction costs of skid trails and might be more suitable for large scale strategies using steep slopes to deter elephants. Costs of such large-scale strategies using skid trail construction must be assessed for their feasibility.

The estimated cost of skid trail construction is approximately $US 3,000/km [46,47]; but it is much less expensive than the construction of electric fences, the most effective known deterrent, which costs approximately $US 8,000/km [48]. Electric fences also require significant maintenance, including the clearing of large trees so they do not fall onto the fence, removal of branches and vegetation that could short the fence, and a reliable energy source [48,49]. Moreover, elephants can quickly learn to use their tusks to break down fences without being shocked, and electric fences killed 80 elephants in India over 12 years [48–52]. Skid trails do not present these same disadvantages. Even so, farming on steep hillsides and constructing skid trail walls also have their challenges, including lack of available hillside area for farming, erosion and loss of soil fertility [53]. Further research is needed to evaluate the practicality and sustainability of farming on steep slopes, or perhaps using terraces to minimize erosion and maximize production on slopes. The feasibility of farming on steep fields needs also to be compared with other methods, such as the use of beehives [31,54,55] and chili pepper [56,57] which have recently received attention for reducing human-elephant conflict using small (i.e. farmer level) and large (i.e. landscape level) scale approaches. Testing innovative strategies to reduce human-elephant conflict is essential to securing the livelihoods of rural people and the existence of forest elephants.

## Supporting information

S1 Table Description of the 39 explanatory variables describing characteristics of farmers, plantations, farming practices and crops. SWS = short wet season, MWS = main wet season, SDS = short dry season, MDS = main dry season. Distances were determined based on GIS coordinates. Types of elephant deterrents are: Type1 = fences with sheet metals; Type2 = wire fences; Type3 = Wire fences with or without noisemakers, plus empty barrels, Type4 = wire fences, plus empty barrels, plus fire, Type5 = others deterrent mixes. In traditional slash and burn agriculture (TSBA) in Gabon seven main tasks or activities related to seven steps are performed before harvesting crops: removing small vegetation, cutting trees, burning dried vegetation, cleaning the area from trunks left after vegetation burning, protecting fields with deterrents, planting crops and cleaning areas from undesired vegetation. Apart from the first three activities, the others are performed randomly according to farmer feeling of priority.
(DOCX)
