# Supplementary material for: Do topography and fruit presence influence occurrence and intensity of crop-raiding by forest elephants (Loxodonta africana cyclotis)?
Source: PLoS One. 2019 Mar 22;14(3):e0213971. doi: 10.1371/journal.pone.0213971 (PMC6430389; doi:10.1371/journal.pone.0213971)
Supplement: S1 Table — SWS = short wet season, MWS = main wet season, SDS = short dry season, MDS = main dry season. Distances were determined based on GIS coordinates. Types of elephant deterrents are: Type1 = fences with sheet metals; Type2 = wire fences; Type3 = Wire fences with or without noisemakers, plus empty barrels, Type4 = wire fences, plus empty barrels, plus fire, Type5 = others deterrent mixes. In traditional slash and burn agriculture (TSBA) in Gabon seven main tasks or activities related to seven steps are performed before harvesting crops: removing small vegetation, cutting trees, burning dried vegetation, cleaning the area from trunks left after vegetation burning, protecting fields with deterrents, planting crops and cleaning areas from undesired vegetation. Apart from the first three activities, the others are performed randomly according to farmer feeling of priority. (DOCX) [file pone.0213971.s001.docx]

# Supporting information

# S1 Table: Description of the 39 explanatory variables describing characteristics of farmers, plantations, farming practices and crops.

| **Characteristics of farmers** | **Characteristics of plantations** | **Farming practices** | **Data on crops** |
| --- | --- | --- | --- |
| Gender  (Male, Female) | NTFP trees species (*Pseudospondias microcarpa, Chrysophyllum africanum, Irvingia gabonensis and Tetrapleura tetraptera*) | Setting seasons of plantations  (SWS, MWS, SDS, MDS) | Crops present  (Banana, Cassava, Sweet Potato, Cocoyam, Sugarcane, Pineapple, Corn, Cucumber, Garden egg, Amaranth, Roselle) |
| Residence status  (Yes, No) | Distance from farms to villages (<0.5km, >0.5km) | Farming task steps | Crop abundance (High, Low) |
| Age  (Adult, Old) | Presence of fruit trees  (Yes, No) | Seasons of task (activity) implementation  (SWS, MWS, SDS, MDS) | Harvest time  (Months) |
| Education  (None, Primary school, Secondary school, University) | Position of fruit trees  (Inside or Outside of field but within 50m) | Crop planting (or seed sowing) steps | Crop growth stages (Vegetative, Flowering, Fruiting, Senescent) |
| Number of family members  (<5, >5) | Fruiting seasons  (SWS, MWS, SDS, MDS) | Crop planting (or seed sowing) seasons  (SWS, MWS, SDS, MDS) | Occurrence of elephant crop damage  (Yes, No) |
| Number of revenue sources  (1, 2, etc.) | Size of field (Small, Average, Large) | Seasons of elephant crop damage  (SWS, MWS, SDS, MDS) | Number of elephant raiders  (<5 or >5) |
| Number of fields (1, 2, etc.) | Field topography  (Flat, Shallow or Steep) | Deterrent used (Scarecrow, Fire, Fence, Empty barrels) | Frequency of crop damage  (Rare, Sometimes, Always) |
| Order of importance of revenue resources  (1^st^, 2^nd^, etc.) | Distance from field to MCNP border  (>10km, <10km) | Effectiveness of deterrent used  (Effective, Not effective) | Extent of damage on crops (Major, Moderate, Minor) |
|  | Distance from farms to MCNP Buffer zone  (>5km, <5km) | Presence of employees  (Yes, No) | Crop classification according to level of damage |
|  | Distance from field to elephant trails  (Near, Far) |  |  |
|  | Presence of elephant deterrents (Yes, No) |  |  |
|  | Type of deterrents  (Type1,Type2, Type3, Type4, Type5) |  |  |
|  | Start of fruiting  (Month of year) |  |  |

SWS = short wet season, MWS = main wet season, SDS = short dry season, MDS = main dry season. Distances were determined based on GIS coordinates. Types of elephant deterrents are: Type1 = fences with sheet metals; Type2 = wire fences; Type3 = Wire fences with or without noisemakers, plus empty barrels, Type4 = wire fences, plus empty barrels, plus fire, Type5 = others deterrent mixes. In traditional slash and burn agriculture (TSBA) in Gabon seven main tasks or activities related to seven steps are performed before harvesting crops: removing small vegetation, cutting trees, burning dried vegetation, cleaning the area from trunks left after vegetation burning, protecting fields with deterrents, planting crops and cleaning areas from undesired vegetation. Apart from the first three activities, the others are performed randomly according to farmer feeling of priority.
